# Pre- and Post-Operative Online Prediction of Outcome in Patients Undergoing Endovascular Coiling after Aneurysmal Subarachnoid Hemorrhage: Visual and Dynamic Nomograms

**DOI:** 10.3390/brainsci13081185

**Published:** 2023-08-10

**Authors:** Zhou Zhou, Fusang Wang, Tingting Chen, Ziqiao Wei, Chen Chen, Lan Xiang, Liang Xiang, Qian Zhang, Kaizong Huang, Fuping Jiang, Zhihong Zhao, Jianjun Zou

**Affiliations:** 1 Department of Clinical Pharmacology, Nanjing First Hospital, Nanjing Medical University, Nanjing 210000, China; evanzhow_0826@163.com (Z.Z.); chenchen266@sina.com (C.C.); zhangq0619@163.com (Q.Z.); kzhuang@nju.edu.cn (K.H.); 2 Department of Pharmacy, Sun Yat-Sen Memorial Hospital, Sun Yat-Sen University, Guangzhou 510275, China; wangfusang100@126.com; 3 School of Basic Medicine and Clinical Pharmacy, China Pharmaceutical University, Nanjing 210009, China; 4 The Second Clinical Medicine School of Nanjing Medical University, Nanjing 211166, China; wzq_0312@163.com; 5 Department of Neurology, The First Affiliated Hospital (People’s Hospital of Hunan Province), Hunan Normal University, Changsha 410081, China; 15200805335@163.com (L.X.); 18867201282@163.com (L.X.); 6 Department of Geriatrics, Nanjing First Hospital, Nanjing Medical University, Nanjing 210000, China

**Keywords:** intracranial aneurysm, aneurysmal subarachnoid hemorrhage, endovascular coiling, dynamic nomogram, outcome prediction

## Abstract

Background: Aneurysmal subarachnoid hemorrhage (aSAH) causes long-term functional dependence and death. Early prediction of functional outcomes in aSAH patients with appropriate intervention strategies could lower the risk of poor prognosis. Therefore, we aimed to develop pre- and post-operative dynamic visualization nomograms to predict the 1-year functional outcomes of aSAH patients undergoing coil embolization. Methods: Data were obtained from 400 aSAH patients undergoing endovascular coiling admitted to the People’s Hospital of Hunan Province in China (2015–2019). The key indicator was the modified Rankin Score (mRS), with 3–6 representing poor functional outcomes. Multivariate logistic regression (MLR)-based visual nomograms were developed to analyze baseline characteristics and post-operative complications. The evaluation of nomogram performance included discrimination (measured by C statistic), calibration (measured by the Hosmer–Lemeshow test and calibration curves), and clinical usefulness (measured by decision curve analysis). Results: Fifty-nine aSAH patients (14.8%) had poor outcomes. Both nomograms showed good discrimination, and the post-operative nomogram demonstrated superior discrimination to the pre-operative nomogram with a *C* statistic of 0.895 (95% CI: 0.844–0.945) vs. 0.801 (95% CI: 0.733–0.870). Each was well calibrated with a Hosmer–Lemeshow *p*-value of 0.498 vs. 0.276. Moreover, decision curve analysis showed that both nomograms were clinically useful, and the post-operative nomogram generated more net benefit than the pre-operative nomogram. Web-based online calculators have been developed to greatly improve the efficiency of clinical applications. Conclusions: Pre- and post-operative dynamic nomograms could support pre-operative treatment decisions and post-operative management in aSAH patients, respectively. Moreover, this study indicates that integrating post-operative variables into the nomogram enhanced prediction accuracy for the poor outcome of aSAH patients.

## 1. Background

Aneurysmal subarachnoid hemorrhage (aSAH) is a highly aggressive subtype of acute stroke that leads to loss of life in approximately 30% of patients, with 50% of survivors potentially unable to regain full functional independence [1,2,3]. Unfortunately, aSAH is prone to occur in adulthood (median age 55) when an individual in the general population is likely to have a good life expectancy and be at the height of their careers [4,5]. Despite advances in aneurysm repair techniques, with endovascular coiling and neurosurgical clipping surgery as the two most common treatment options, both have the potential to induce neurological deterioration and disappointing long-term quality of life. A meta-analysis study reported that 24% of patients undergoing coiling and 32% undergoing surgical clipping still had unfavorable functional outcomes at 1-year follow-up [6]. In addition, another study showed a lower risk of seizures 1 year after embolization compared with clipping, but the incidence of late rebleeding may be relatively high [7].

It is hypothesized that physicians can forecast whether an aSAH patient could regain functional independence after treatment. In this case, discussing the expected outcome with patients, therefore guiding individualized decisions about treatment modality and neurological care, would be very beneficial to improve the patient’s recovery potential and long-term effects. Therefore, there is an urgent need to develop two predictive models that could support pre-operative and post-operative clinical decision-making in different disease courses.

In this context, several prediction models [8,9,10] based on only pre-operative variables have been developed to predict the functional outcome of aSAH patients. However, these models could have clinical utility only in the pre-treatment decision-making because post-operative complications (vasospasm, rebleeding, intracranial infection, delayed cerebral ischemia, hydrocephalus, etc.) largely influence the functional outcome in the clinical course which were not included in these models [11,12,13]. In addition, a machine learning model, including post-operative complications, was also developed in a previous study to predict the functional outcome at discharge [14]. Nevertheless, the fact that it could not be visualized as an easy-to-use bedside prediction tool limits its clinical application. Therefore, previous conventional predictive models were unable to provide a comprehensive and systematic prognostic assessment of the perioperative patient as well as to guide reasonable clinical treatment.

In the present study, we aimed to develop two models based on accessible feature variables for the pre-operative and post-operative prediction of the 1-year functional outcome in Chinese aSAH patients undergoing endovascular coiling, respectively, and visualize the prediction tools as easier-to-use web-based risk calculators with greater clinical applicability.

## 2. Methods

### 2.1. Study Population

This study was reviewed and approved by the Ethics Committee of Hunan Provincial People’s Hospital (document number: [2015]-10). To develop the predictive model, we retrospectively analyzed the data of consecutive aSAH patients admitted to the neurology department of People’s Hospital of Hunan Province (China) between November 2015 and December 2019. Only patients who met the following inclusion criteria: age ≥ 18 years, rupture of the aneurysm as the cause of SAH confirmed by three-dimensional computed tomographic angiography (CTA), magnetic resonance angiography (MRA), or digital subtraction angiography (DSA), patients with intracranial saccular aneurysms who underwent specialized endovascular coil embolization, including the use of stent-assisted coiling, and available modified Rankin Scale (mRS) at 1-year follow-up. Patients with previous aneurysm repair, medical history of SAH or stroke, non-aneurysmal SAH, or missing data were excluded.

### 2.2. Treatment of Ruptured Aneurysm

Depending on the characteristics of patients and aneurysms, the decision to adopt coiling or clipping, or other clinical treatment options was made jointly by cerebrovascular neurosurgery and neurology specialists. When the ruptured aneurysm was suitable for coiling, unable to benefit from clipping, or located in the posterior circulation (PC) artery, more consideration should be given to endovascular treatment, which was widely recommended as a safer and reliable option. In addition, stent-assisted coiling was considered for aneurysms with a wide neck or dissecting aneurysms. All patients who underwent stent-assisted coiling received dual antiplatelet therapy (aspirin and clopidogrel) to prevent in-stent thrombosis and subsequent ischemic events as much as possible.

### 2.3. Data Collection and Definition

On admission, the following data were obtained from patients: demographic data (gender and age), smoking history, drinking history, medical history (hypertension, diabetes, hyperlipidemia, coronary heart disease, and atrial fibrillation), clinical score on admissions (Glasgow Coma Scale [GCS] score, and Hunt-Hess grade), and morphologic characteristics (aneurysm size, location, shape, multiplicity, and neck width). The GCS score was the combined result of a three-part behavioral (eye, motor, and verbal) response assessment, and each was evaluated independently [15]. If a patient was already intubated on admission, it may not be possible to assess verbal response. For this, a validated extrapolation tool was taken into account to impute the total GCS score [16]. A wide neck was defined as a neck size ≥ 4 mm or dome-neck ratio ≤ 2. The aneurysmal shape was classified as regular and irregular (defined as the presence of aneurysm wall protrusions, bi- or multi-lobular, or small blebs). Aneurysmal location was divided as anterior circulation versus PC location. 

Primary post-operative complications included cerebral vasospasm (CVS), delayed cerebral infarction (DCI), rebleeding, and hydrocephalus, intracranial infection, respiratory tract infection (RTI). CVS was defined as radiologically confirmed vasoconstriction on DSA, CTA, or MRA. DCI was defined as new infarcts on CTA or MRA, not including infarction within 48 h after treatment. Rebleeding was a new or expanded hemorrhage from an aneurysm on CTA. Hydrocephalus was defined as radiographically confirmed ventriculomegaly. Symptomatic hydrocephalus with drowsy or decreased GCS and so on was intervened by external ventricular drainage (EVD). The result of immediate angiographic occlusion was assessed based on the Raymond grade, and a Raymond grade of I denoted complete occlusion.

### 2.4. Outcome Measure

Our outcome measure was 1-year mRS scores which were used to quantify the degree of independence in patients’ activities of daily living. Six categories were on this order scale, 0 = no symptoms, 1 = no significant disability despite symptoms, 2 = slight disability, 3 = moderate disability, 4 = moderately severe disability, 5 = severe disability, and 6 = death. Functional outcome was dichotomized into good outcome (mRS 0–2) and poor outcome (mRS 3–6), and consequently study participants were divided into two groups and formed the cohort control.

### 2.5. Statistical Analysis and Construction of Dynamic Nomogram

Statistical analyses were conducted using SPSS software (version 25.0, IBM Corporation, Armonk, NY, USA) and R version 4.1.0 (http://www.R-project.org/, accessed on 6 June 2023). All tests were two-sided, and *p* < 0.05 was a statistical significance level. To explore the additional values of post-operative variables to outcome prediction in aSAH patients, we developed two prediction models based on baseline variables with/without post-operative compilations. Univariate and multivariate analyses were used to select the best predictors of a 1-year poor outcome. The association of each variable with 1-year outcome was first tested by univariate analyses (Mann–Whitney U test, Student *t*-test, or Chi-squared test, when appropriate).

Furthermore, multivariate logistic regression analysis was performed to use the variables with a *p*-value < 0.1 in the univariate analysis as independent variables and 1-year poor outcome as a dependent variable. Finally, after a forward stepwise model selection procedure based on the Akaike information criterion, the independent predictors entered into the final regression were used to construct a graphic nomogram based on the “RMS” package of R software. Eventually, we used the “DynNom” package to convert the graphic nomogram into a dynamic nomogram, an online calculator conveniently available on the web.

### 2.6. Validation of Model

Internal validation was performed based on 1000 bootstrap resamples. Model performance was assessed in three domains: discrimination, calibration, and clinical utility. The discrimination ability (the ability to discriminate between good and poor outcomes) was quantitatively evaluated by calculating the *C* statistic, sensitivity, and specificity. The Hosmer–Lemeshow test and calibration plots were used to assess the calibration capability statistically and graphically, a measure of how close the predicted probabilities are numerically to the actual results, respectively. Finally, the expected net benefits of the nomogram model for different threshold probabilities were quantified with the aid of decision curve analysis (DCA) [17,18]. The true-positive rate and false-positive rate classifications were considered at increasing decision thresholds, where the net benefit = true-positive rate − false-positive rate × threshold probability/(1 − threshold probability).

## 3. Results

### 3.1. Patient Characteristic

**Figure 1:**
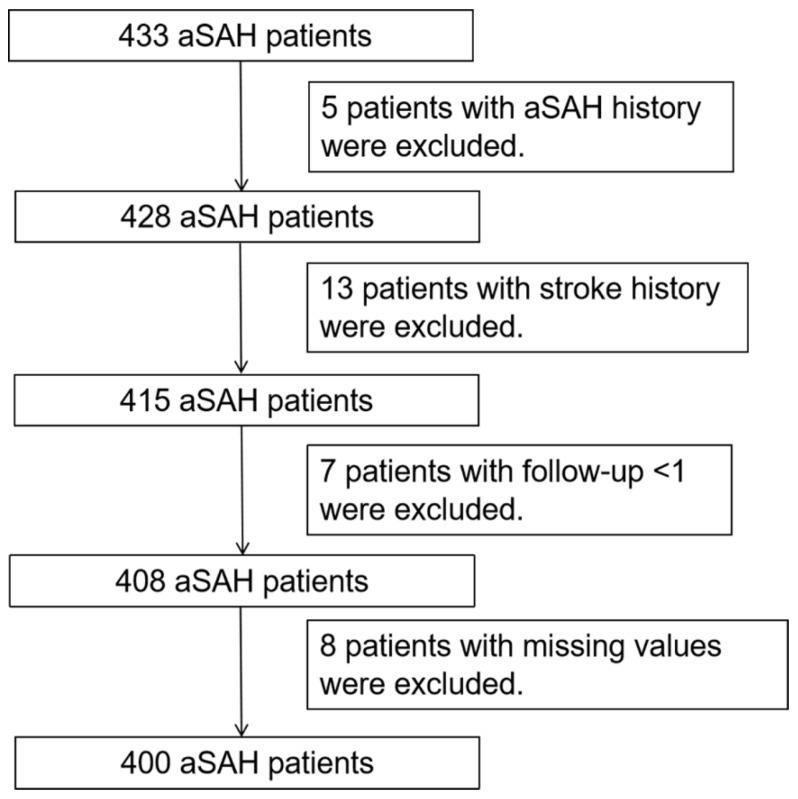
Flow chart of patient inclusion and exclusion.

The flow chart of patient inclusion and exclusion is shown in Figure 1. A total of 400 aSAH patients were included in the present analysis (mean age ± SD 58.6 ± 10.7) including 122 (30.5%) males and 278 (69.5%) females (Table 1). Of these, 14.8% (59/400) suffered from functional dependence or death (i.e., poor outcome) at 1-year follow-up. Furthermore, after intravascular treatment, the prevalence of aneurysm rebleeding was 6.75% (27/400), CVS was 24% (96/400), hydrocephalus was 4.5% (18/400), DCI was 12.25% (49/400), intracranial infection was 1.5% (6/400), and RTI was 29.75% (119/400) (Table 2).

### 3.2. Variable Selection (by Univariate and Multivariate Analyses)

In univariate analyses, age (*p* < 0.001), Hunt-Hess grade on admission (*p* < 0.001), GCS score on admission (*p* < 0.001), aneurysms size (*p* < 0.03), location (*p* < 0.008), aneurysm rebleeding (*p* < 0.001), CVS (*p* < 0.001), hydrocephalus (*p* < 0.001), intracranial infection (*p* = 0.061), DCI (*p* < 0.001), and RTI (*p* < 0.001) was significantly associated with functional outcome (Table 1 and Table 2).

The stepwise regression analysis based on only baseline characteristics identified age, GCS score on admission, and PC location as the independent predictors for poor outcomes in aSAH. In previous studies, aneurysm size was the independent predictor of poor outcomes in aSAH [19,20]. Aneurysm size was also incorporated into the pre-operative prediction model. By multivariate logistic regression, the associations of the 4 predictors with poor outcome was given as odds ratios (ORs) with 95% confidential intervals (CIs): age (OR: 1.078, 95% CI: 1.044–1.115, *p* < 0.001), aneurysm size (OR: 1.075, 95% CI: 0.939–1.224, *p* = 0.285), GCS score on admission (OR: 0.795, 95% CI: 0.731–0.862, *p* < 0.001), PC location (OR: 6.188, 95% CI: 2.097–17.670, *p* < 0.001, Table 3).

The stepwise regression analysis based on both baseline characteristics and post-operative variables derived 9 independent predictors (Table 3): PC location (OR: 4.520, 95% CI: 1.239–16.448, *p* = 0.021), age (OR: 1.074, 95% CI: 1.035–1.118, *p* < 0.001), aneurysm size (OR: 1.120, 95% CI: 0.968–1.296, *p* = 0.124), GCS score on admission (OR: 0.891, 95% CI: 0.806–0.987, *p* = 0.026), rebleeding (OR: 8.103, 95% CI: 2.779–24.050, *p* < 0.001), hydrocephalus (OR: 3.462, 95% CI: 0.937–12.707, *p* = 0.059), DCI (OR: 3.843, 95% CI: 1.539–9.872, *p* = 0.004), CVS (OR: 2.933, 95% CI: 1.311–6.569, *p* = 0.008), and RTI (OR: 1.982, 95% CI: 0.911–4.297, *p* = 0.082). Rebleeding was the most significant risk factor for poor outcome, followed by PC location, DCI, hydrocephalus, CVS, and RTI.

### 3.3. Construction and Performance of Visualized Online Nomograms

#### Development of Nomograms

**Figure 2:**
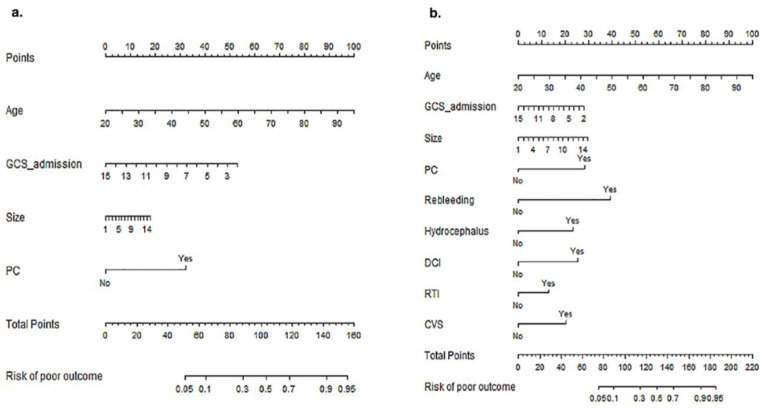
The pre-operative (**a**) and post-operative graphic nomograms (**b**) of 1-year poor outcome of aneurysmal subarachnoid hemorrhage.

**Figure 3:**
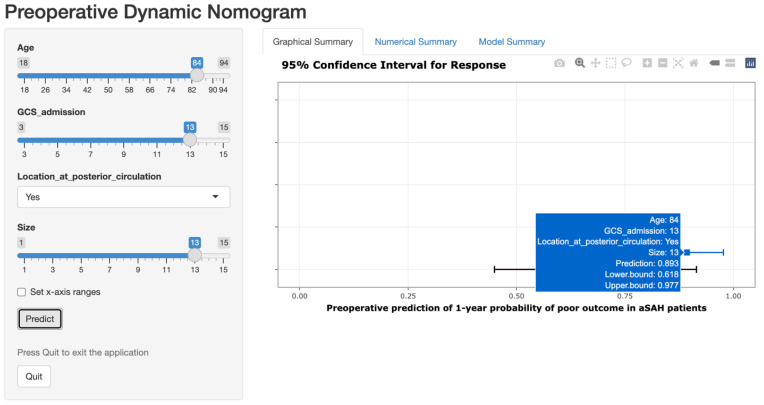
An example of using the pre-operative dynamic nomogram to predict the 1-year poor outcome of aneurysmal subarachnoid hemorrhage.

According to the results of logistic regression analysis, the pre-operative model was derived from the following logistic regression equation: Log [p(x)/1 − p(x)] = −3.943 + age × 0.075 + size × 0.072 − GCS score on admission × 0.230 + PC × 1.823, where the p(x) is the probability of poor outcome. The post-operative model results: Log [p(x)/1 − p(x)] = −6.769 + age × 0.071 + size × 0.113 − GCS score on admission × 0.115 + location at PC × 1.508 + Rebleeding × 2.092 + Hydrocephalus × 1.242 + DCI × 1.346 + CVS × 1.076 + RTI × 0.684, where the p(x) is the probability of poor outcome. As shown in Figure 2, the two statistical models were visualized as graphic nomograms. Furthermore, to facilitate the clinical application, we developed two web-based dynamic nomograms, which were accessible for free on https://preoperativenomogram.shinyapps.io/DynNomapp/ (accessed on 4 August 2023) and https://postoprativenomogram.shinyapps.io/DynNomapp/ (accessed on 4 August 2023). Figure 3 shows an example of the dynamic nomogram to predict the 1-year poor outcome of aneurysmal subarachnoid hemorrhage.

### 3.4. Discrimination and Calibration of Nomograms

**Figure 4:**
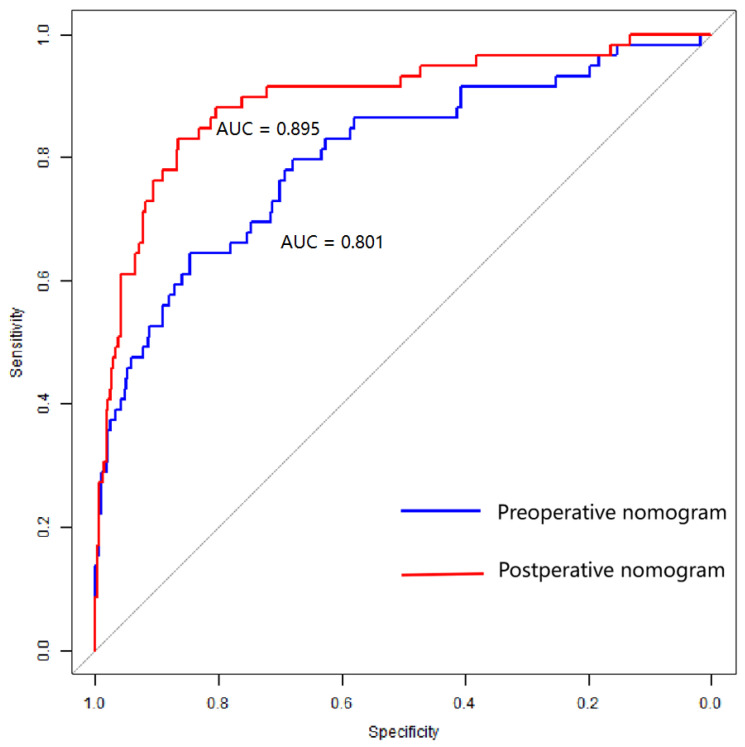
Receiver operating characteristic (ROC) curves of the pre-operative and post-operative nomograms of 1-year poor outcome of aneurysmal subarachnoid hemorrhage.

**Figure 5:**
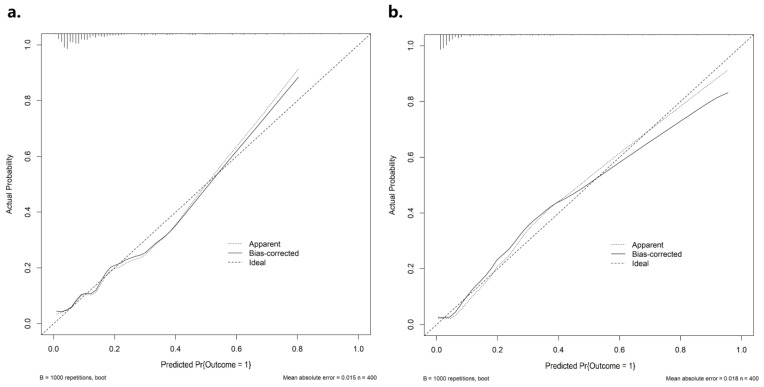
Calibration plots for pre-operative (**a**) and post-operative nomograms (**b**). The horizontal coordinate is the predicted probability; the vertical coordinate is the actual probability. The diagonal dashed line represents the ideal plot of the calibration plot. The dotted line represents the performance of the nomogram, while the solid line corrects for any bias in the nomogram.

The pre-operative nomogram based on pre-operative variables (age, aneurysms size, GCS score on admission, and PC location) demonstrated good discrimination with a *C* statistic of 0.801 (95% CI: 0.733–0.870), sensitivity of 0.678 and specificity of 0.880. By adding post-operative complications (rebleeding, hydrocephalus, DCI, CVS, and RTI) to the post-operative nomogram, the discriminatory ability of the model to predict a 1-year poor outcome was significantly improved, with a *C* statistic increasing to 0.895 (95% CI: 0.844–0.945, *p* < 0.01, Figure 4), and its sensitivity and specificity were 0.658 and 0.922, respectively. The Hosmer–Lemeshow test demonstrated that the pre-operative (χ^2^ = 7.365, *p* = 0.498) and post-operative nomogram (χ^2^ = 9.845, *p* = 0.276) were well calibrated. Moreover, the calibration curves indicated that the nomogram predicted the probability of poor outcome were in good agreement with the actual likelihood, i.e., the incidence of poor prognosis in the real world (Figure 5). 

### 3.5. Clinical Utility of Nomograms

**Figure 6:**
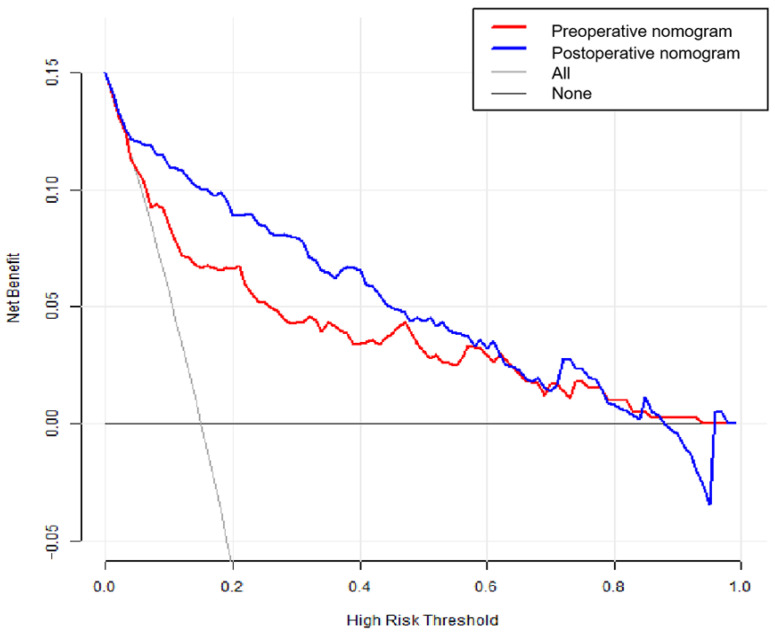
Results of decision cure analysis for the pre-operative and post-operative nomograms.

In the DCA plot, the “High-Risk Threshold” of the horizontal coordinate is threshold probability, a threshold to determine whether the patient should be treated. If the model’s predicted probability is higher than the threshold probability, then the patient is regarded as a “positive case” and should be treated. Conversely, the patient would be defined as a “negative case” and not be treated. For example, a risk threshold of 40% is chosen, which means that if the probability of a 1-year poor outcome is >40%, the patient will undergo some therapeutic intervention. It is clear from the analysis in Figure 6 that when the threshold probabilities of poor outcome range from 10% to 90%, using nomograms to predict poor outcomes in aSAH generates more net benefit than “treat all” or “treat none” strategies. Although there are some overlaps, it must be acknowledged that the post-operative nomogram produces a greater net benefit than the pre-operative one.

## 4. Discussion

To our knowledge, this is the first attempt to construct two dynamic nomograms that can be used to predict 1-year functional outcomes of aSAH patients preoperatively and postoperatively. The pre-operative nomogram included age, size, location, and GCS score on admission, and the post-operative nomogram included these predictors as well as post-operative rebleeding, hydrocephalus, CVS, DCI, and RTI. Both nomograms had good discrimination (*C* statistic = 0.895 [95% CI: 0.844–0.945] vs. 0.801 [95% CI: 0.733–0.870]) and calibration (χ^2^ = 7.365 vs. 9.845). Moreover, our study reflected the satisfactory added value of post-operative complications in predicting poor outcomes compared with the pre-operative model.

One strength of our study is that the two nomograms constructed could provide outcome predictions in the different disease courses. Specifically, the pre-operative nomogram can help guide which patients are not suitable for endovascular coiling, and the post-operative model can assist in guiding which patients should receive closer clinical monitoring and more timely interventions. Both prediction tools are beneficial to reducing the risk of post-operative 1-year poor outcome. We also demonstrated that the post-operative nomogram had better performance (*C* statistic = 0.895) than the pre-operative nomogram (*C* statistic = 0.801), which suggested comprehensive and integrated predictive modeling of baseline data, intraoperative and post-operative factors, and complications for more accurate predictions. 

Another strength of our study was that we visualized the prediction models as online calculators, namely dynamic nomograms, which means that clinicians and patients can visit the website quickly with just a smartphone or computer to predict the prognosis conveniently and efficiently for each patient. Nomogram is widely recommended as a visual statistical tool that provides an easy-to-understand and attractive graphical version of logistic regression models, reflecting individual precise prognostic risk probabilities, therefore supporting clinical decision-making [21,22,23]. However, as the models encompass many variables and become more complex, the use of traditional nomograms in static graphical versions can become cumbersome. The advantages of the dynamic nomograms are then highlighted, eliminating the need to manually measure the effect size of each variable as with a traditional nomogram. By creating a user-friendly electronic interface, it is possible to automatically visualize the final summed predicted values and corresponding confidence intervals, making it easy for observers to interact with the prediction model in real time [24]. As a specific example, the nomogram assigns about a 90% probability of poor outcome in an aSAH patient aged 84 years (84 points) after endovascular coiling, who was admitted with a GCS score of 13 (8 points) and a 13mm aneurysm (18 points) that was in the PC (31 points), resulting in a total score of 141 points. The corresponding web-based nomogram is shown in Figure 3, where age, GCS score on admission, and aneurysm size were slid to 84, 13, and 13, and the location of the PC was selected (yes), and finally “predict” was clicked, which automatically output a predicted probability of 0.893. In the future, our nomograms could ideally be combined with the hospital’s EHR system to enable the automatic extraction of real-time patient data and dynamically trigger early-warning signals that indicate the requirement for targeted monitoring of risk factors.

Four pre-operative predictors (i.e., age, GCS score on admission, aneurysm size, and location) were included in the pre-operative nomogram and were retained in the post-operative nomogram. Age was the most significant predictor in the pre-operative nomogram. Age was positively associated with 1-year poor outcome after aSAH, which was underpinned by numerous studies. For instance, age was incorporated as a positive predictor into the modeling of poor outcome prediction after aSAH in the study of Blessings et al. [9] and Liu et al. [25]. Moreover, Carlina et al. found that age > 60 was a risk factor for poor outcomes after aSAH [10]. The GCS score has been widely accepted as a common grading system suitable for assessing the level of consciousness. It has become the standard assessment tool for traumatic brain injury [26]. In the current study, we confirmed that a lower GCS score on admission was correlated with a higher risk of poor outcomes at 1 year. The relationship between the two has been described in detail in several previous studies [25,27]. Given the research reported by Oshiro and colleagues [28], there is confidence that the GCS score may have equal or even greater predictive value compared to the WFNS and Hunt and Hess grades. The aneurysmal size was also positively linked to poor outcomes of aSAH patients, and aneurysmal size ≥ 20 mm was reported as a risk factor for poor outcomes [9,29]. PC location has already been significantly linked with poor outcome risk, which was consistent with the result of our study [30]. However, although a study indicated that PC location had a negative association with poor outcome risk, the sample size of this study was quite small [8]. Therefore, there is a need for future studies with large samples to investigate further the relationship between PC location and poor outcome risk after aSAH. Furthermore, we found that the final pre-operative model did not include the admission laboratory tests underlined by Li et al. [31]. Although the discrimination ability was not seriously affected in comparison, it deserves to be explored in a targeted way.

Also, it was observed that five post-operative complications, rebleeding, hydrocephalus, CVS, DCI, and RTI, are more likely to contribute to the poor prognosis of aSAH one year later. These secondary complications during the in-hospital course were all devastating complications usually experienced by aSAH patients. Hostettler et al. developed a score consistent with our study to predict 1-year functional outcome after aSAH, with rebleeding and hydrocephalus as two predictors [32]. Rebleeding was strongly tied with worse functional outcomes [33]. Another study documented hydrocephalus as a risk factor for poor outcomes [34]. CVS was a predictor of 1-year poor outcome after aSAH in our study. This was supported by a meta-analysis that observed that around one third of aSAH patients with CVS would have poor outcome [35]. The narrowed blood vessels in the subarachnoid space of the brain resulting from CVS could further cause DCI and then progress to cerebral infarction [36]. This was also in line with our result that the occurrence of cerebral infarction would increase the risk of poor outcomes. Cerebral infarction had a close correlation with functional outcomes. A study implied this kind of causality and claimed that decreased frequency of cerebral infarction could improve functional outcomes after aSAH [37]. Additionally, van der Harst et al. [38] have recently published results suggesting that Near-infrared spectroscopy regional cerebral tissue oxygen saturation (NIRS rSO2) monitoring could improve the accuracy of determining whether a patient has suffered DCI compared to DSA, CTA, and other modalities. It will be possible in the future to combine this measurement technology with our developed post-operative models to facilitate the identification of high-risk patients. Our study showed that RTI was related to poor outcomes. A few studies have reported that post-operative pneumonia was a risk factor for poor outcomes [34,39]. Therefore, we recommended the effective prevention and aggressive treatment of RTI to improve the prognosis of aSAH patients.

## 5. Limitations

Some limitations should be acknowledged. First, since this is a retrospective study, the performance of models needs prospective verification. Second, the database utilized in the study was collected from the Department of Neurology of a tertiary center in China and there was a lack of information on patients who underwent surgical clipping. Currently, in clinical practice, the indications for endovascular treatment are gradually increasing, considering the ability of endovascular coils to yield less symptomatic vasospasm and higher disability-free survival rates. It has become a highly promising therapy recommended for the clinical management of ruptured aneurysms [40,41,42], with approximately 80% of aneurysm patients undergoing coiling rather than surgical clipping [6]. It is, therefore, reasonable and understandable that the database would not contain information on patients who have undergone clipping. Moreover, there may be no disadvantage in limiting the study population to aSAH patients treated with a particular therapy. A predictive model focused on the population undergoing endovascular coiling may generate a more accurate and individual prediction for the prognosis of this population, therefore improving the quality of survival for the entire community to a greater extent. Likewise, a recent study included only aSAH patients receiving clipping [29]. It should also be acknowledged, however, that the models developed should be cautiously applied to patient groups with different treatment plans, such as open surgery and flow diverters. Thirdly, our pre- and post-operative models do not incorporate radiological indicators such as Fisher grade that could demonstrate computed tomography (CT) findings, considering that Fisher grade is typically used to assess the risk of vasospasm [43] rather than specifically to predict long-term functional recovery of patients after treatment, we have made a more targeted replacement for it. The final models developed successfully covered several post-operative complications including CVS and DCI, and demonstrated excellent predictive performance. This precisely confirms the finding of Jagger et al. [44] that using neurological sign variables to alternative scales may yield higher accuracy in predicting outcome. A possible explanation is that the interobserver reliability of these scales has proven to be low presently because of the CT results and ambiguous grading definitions alone [45,46,47,48]. Moreover, the studies by Zheng et al. [27] and Shirao et al. [49] reported that the Fisher scale failed to show an independent correlation with favorable prognosis in multivariate analysis. Therefore, the lack of Fisher grade in the nomogram is understandably acceptable. 

Finally, our models do not replace experienced clinicians to independently perform bedside diagnostic and therapeutic decision-making but rather act as a clinical supportive “early-warning” tool. Specifically, once a patient suddenly becomes symptomatic, even in the non-acute phase, clinicians can immediately open the web-based risk calculators to perform risk stratification and provide rational interventions in conjunction with clinical experience to maximize the long-term favorable prognosis for him. Thus, further work should include external validation of models through multicenter studies to enhance reliable stability during clinical usage. 

## 6. Conclusions

Overall, both nomograms we developed had excellent performance and perfect clinical utility, and two could support pre-operative and post-operative clinical decision-making, respectively. To be specific, the pre-operative model can help clinicians determine whether an aSAH patient is appropriate for endovascular coiling, while the post-operative model can help guide which patients should be given more timely intervention management and more accurately predict functional outcomes 1 year after endovascular treatment. Also, by introducing online web-based calculators, risk scores can be measured more easily and visually, therefore facilitating clinical application. In the future, we will attempt to extend the constructed models to other acute-onset cerebrovascular events, such as ischemic stroke, apart from external validation in multicenter cohorts, to examine their ability to perform in different populations.

## Figures and Tables

**Table 1:** Result of univariate analysis of baseline characteristics.

Baseline Characteristics	Total Cohort (n = 400)	Good Outcome (n = 341)	Poor Outcome (n = 59)	*p*-Value
Demographic data				
Age, years, mean (SD)	58.6 (10.7)	57.30 (10.09)	65.88 (11.25)	<0.001 ^†^
Gender (Female), n (%)	278 (69.5)	101 (29.6)	21 (35.6)	0.443
Medical history, n (%)				
Hypertension	201 (50.3)	166 (48.7)	35 (59.3)	0.171
Diabetes mellitus	15 (3.8)	12 (3.5)	3 (5.1)	0.831
Hyperlipidemia	4 (1.0)	3 (0.9)	1 (1.7)	1.000
Coronary heart disease	30 (7.5)	23 (6.7)	7 (11.9)	0.267
Atrial fibrillation	4 (1.0)	3 (0.9)	1 (1.7)	1.000
Smoking, n (%)	64 (16.0)	55 (16.1)	9 (15.3)	1.000
Drinking, n (%)	25 (6.3)	21 (6.2)	4 (6.8)	1.000
Clinical status on admission				
Hunt-Hess grade on admission, n (%)				<0.001 ^†^
Ⅰ	29 (7.3)	26 (7.6)	3 (5.1)	
Ⅱ	278 (69.5)	256 (75.1)	22 (37.3)	
Ⅲ	55 (13.8)	39 (11.4)	16 (27.1)	
Ⅳ	38 (9.5)	20 (5.9)	18 (30.5)	
GCS on admission, mean (SD)	13.7 (2.93)	14.21 (2.20)	10.93 (4.62)	<0.001 ^†^
Aneurysmal characteristics				
Aneurysmal size, mm, n (%)	5.04 (2.22)	4.94 (2.10)	5.62 (2.72)	0.030 ^†^
Multiple aneurysm (yes), n (%)	115 (28.8)	95 (27.9)	20 (33.9)	0.429
PC location (yes), n (%)	22 (5.5)	14 (4.1)	8 (13.6)	0.008 ^†^
Irregular shape (yes), n (%)	217 (54.3)	184 (54.0)	33 (55.9)	0.889
Wide neck (yes), n (%)	289 (72.3)	245 (71.8)	44 (74.6)	0.784

Abbreviations: GCS = Glasgow Coma Scale, PC = posterior circulation, SD = standard deviation. ^†^ Indicates significance (*p* < 0.05).

**Table 2:** Disease Management, immediate angiographic results, and post-operative complications.

Variables	Total Cohort (n = 400)	Good Outcome (n = 341)	Poor Outcome (n = 59)	*p*-Value
Embolization technique, n (%)				0.268
Coiling only	179 (44.8)	157 (46.0)	22 (37.3)	
Stent-assisted coiling	221 (55.3)	184 (54.0)	37 (62.7)	
Immediate aneurysm occlusion, n (%)				0.925
Complete occlusion	387 (96.8)	329 (99.4)	58 (98.3)	
Incomplete occlusion	3 (0.8)	2 (0.6)	1 (1.7)	
Aneurysm rebleeding, n (%)	27 (6.75)	13 (3.8)	14 (23.7)	<0.001 ^†^
Cerebral vasospasm, n (%)	96 (24.0)	60 (17.6)	36 (61.0)	<0.001 ^†^
Hydrocephalus, n (%)	18 (4.5)	7 (2.1)	11 (18.6)	<0.001 ^†^
EVD, n (%)	13 (3.25)	4 (1.2)	9 (15.3)	<0.001 ^†^
DCI, n (%)	49 (12.3)	25 (7.3)	24 (40.7)	<0.001 ^†^
Intracranial infection, n (%)	6 (1.5)	3 (0.9)	3 (5.1)	0.061
RTI, n (%)	119 (29.8)	79 (23.2)	40 (67.8)	<0.001 ^†^

Abbreviations: CVS = cerebral vasospasm, EVD = external ventricular drainage, DCI = delayed cerebral infarction, RTI = respiratory tract infection, SD = standard deviation. ^†^ Indicates significance (*p* < 0.05).

**Table 3:** Pre-operative and post-operative predictors of functional outcome.

Predictors	Pre-Operative Model			Post-Operative Model		
	OR	95% CI	*p*-Value	OR	95% CI	*p*-Value
Age	1.078	1.044–1.115	<0.001	1.074	1.035–1.118	<0.001
PC location	6.188	2.097–17.670	<0.001	4.520	1.239–16.448	0.021
Size	1.075	0.939–1.224	0.285	1.120	0.968–1.296	0.124
GCS on admission	0.795	0.731–0.862	0.001	0.891	0.806–0.987	0.026
Rebleeding				8.103	2.779–24.050	<0.001
Hydrocephalus				3.462	0.937–12.707	0.059
DCI				3.843	1.539–9.872	0.004
CVS				2.933	1.311–6.569	0.008
RTI				1.982	0.911–4.297	0.082

Abbreviations: OR = Odds Ratio, CI = Confidence internal, CVS = cerebral vasospasm; EVD = external ventricular drainage; DCI = delayed cerebral infarction; RTI = respiratory tract infection.

## Data Availability

The datasets used and/or analyzed during the current study are available from the corresponding author on reasonable request.

## Abbreviations

aSAH, aneurysmal subarachnoid hemorrhage; CTA, computed tomographic angiography; MRA, magnetic resonance angiography; DSA, digital subtraction angiography; mRS, modified Rankin Scale; PC, posterior circulation; GCS, Glasgow Coma Scale; CVS, cerebral vasospasm; DCI, delayed cerebral infarction; RTI, respiratory tract infection; EVD, external ventricular drainage; OR, odds ratios; CI, confidential interval; DCA, decision curve analysis.
